# Optimized Design of Sparse Antenna Array for 2D Subarrays Based on GA-PSO Algorithm and Ambiguity Function

**DOI:** 10.3390/mi16091038

**Published:** 2025-09-10

**Authors:** Jian Yang, Jian Lu, Tong Zhu, Chuanxiang Li, Yinghui Quan

**Affiliations:** 1 School of Engineering, Rocket Force University of Engineering, Xi’an 710025, China; zhutong38173105@163.com (T.Z.); lichuanxiang@zju.edu.cn (C.L.); 2 Unit 96921 of the People’s Liberation Army, Beijing 100020, China; lujian_china@sina.com; 3 School of Information Mechanics and Sensing Engineering, Xidian University, Xi’an 710071, China; yhquan@mail.xidian.edu.cn

**Keywords:** sparse antenna array, 2D subarrays, genetic algorithm–particle swarm optimization (GA–PSO), ambiguity function

## Abstract

A sparse antenna array of subarrays can effectively reduce the digital channels of array antennas, system complexities, and hardware cost while simultaneously increasing the antenna aperture. In this study, a new optimal design is proposed for a sparse antenna array of subarrays in the full-phased multiple input multiple output (FPMIMO) operation mode based on genetic algorithm–particle swarm optimization (GA–PSO) and ambiguity functions. The proposed algorithm can adaptively adjust the number of optimization iterations for yielding the optimization results of the PSO algorithm and GA to ensure the global optimization performance of algorithms and combine ambiguity functions to determine the final optimized sparse antenna array of subarrays. The effectiveness of the proposed algorithm is verified via simulation tests.

## 1. Introduction

In modern radar systems, array antennas are increasing in size, as they contain hundreds or even thousands of antenna elements. As the array antenna becomes large, the digital channels required for each array element remarkably increase the hardware cost and system complexity. Especially in aircraft, ships, and other limited spaces, increasing the array antenna aperture by simply increasing the number of antenna elements is impossible. On the other hand, with the continuous development of antenna array microsystems, low profiles, high efficiency, and light weights are becoming increasingly important, and it is difficult to effectively reduce the hardware overhead of antenna arrays in micro-sized spaces. To overcome these problems, usually a number of array elements are grouped into subarrays to form subarray antennas. To further increase the antenna aperture and enhance the spatial resolution of targets while maintaining the scale, we need to increase the subarray spacing to sparsely arrange them. Therefore, the grouping of array elements into subarrays and arranging them are the primary issues to be solved when designing an antenna array.

Subarray grouping and arrangement are closely related to beam performance. Starting from monopulse angle measurement, Nickel [1] explored methods to fabricate a sum-difference beam with low side lobes and suppressed interference under interference conditions. With regard to subarray designing, Xu et al. [2] used the equivalent noise power method to design nonuniform adjacent subarrays for ULAs with amplitude tapering, low side lobes, and no grating lobes. Because it is impractical to precisely divide the antenna aperture using predefined subarrays, the authors of [3] proposed the concept of relaxation units to develop a mathematical description based on the exact coverage theory, which can obtain a quasi-optimal subarray grouping scheme. Xiong et al. summarized signal processing for phased-array radars (PARs) based on subarrays, analyzed the optimization criterion for subarray grouping, and proposed the research direction for subarray grouping [4]. Zhang et al. [5] proposed a two-multibeam multiplexing design for arbitrary directions with varying and fixed antenna spacings based on the interleaved subarray architecture. In [6], a non-iterative algorithm based on the amplitude density was applied to an initial solution of array sparseness, and the improved GA was used for further optimization, where array element symmetry constraints are proposed to enhance the optimization effect. In [7], a differential evolutionary algorithm was used in the array design to reduce side lobes and the grating lobe level, and then a 2D-MIMO sparse array was designed to achieve high angular resolution in terms of both azimuth and elevation. In [8], an effective approach for synthesizing sparse planar arrays using the three-step mapping method was proposed by decomposing the array optimization process into three steps: region partitioning, placement along the x direction, and placement along the y direction. Because optimization of the subarrays of array antennas is a nonlinear and nonconvex optimization problem, it was achieved by establishing an objective function and finding the optimal solution through the cyclic iterations of intelligent optimization algorithms, such as genetic algorithms [9,10], particle swarm algorithms [11,12], simulated annealing algorithms [13,14], and hybrid algorithms [15,16].

Li et al. proposed a novel array structure comprising irregular and overlapped subarrays [17] which integrated the advantages of two types of subarrays to achieve performance requirements while reducing the cost. Furthermore, several studies have been conducted prior to developing this technology for real application. In one study [18], fine-grid sparse antenna arrays were used to refine traditional half-wavelength grids to improve the freedom of the sparse optimization process and reduce the number of active elements. Furthermore, a new pruned fast Fourier transform was proposed to eliminate all redundant operations in beamforming, one which is suitable for fine-grid sparse antenna arrays. Additionally, a novel sparse antenna array comprising co-prime and nested subarrays was designed, and a corresponding two-dimensional (2D) direction of arrival estimation method was presented [19]. Meanwhile, an irregular subarray-based low-cost sensor array design strategy was also proposed [20]. Especially for large-sized cases, a hierarchical subarray design strategy was presented. A research group [21] considered a shared aperture system where optimum sparse subarrays were allocated to individual SOIs and collectively spanned the entire full array receiver aperture. Each subarray might have its own antenna type and could be composed of different numbers of antennas. An optimum joint sparse subarray design for a shared aperture was proposed based on maximizing the sum of subarray beamformer SINRs, where there are threshold constraints with and without SINRs. Moreover, a multi-objective optimization framework was employed for the placement of subarrays within a prescribed area, which is dictated by form factor constraints [22]. Hence, a tradeoff occurs between the small beam width obtained by spacing out the subarrays and the grating and side lobes created by the sparse placement. Therefore, based on 16-element (4 × 4) subarrays in the 60-GHz band, the effectiveness of the method has been verified via numerical examples.

With the development of multiple-input multiple-output (MIMO) technology, the subarray design is combined with MIMO radar in the hybrid-phased MIMO (HPMIMO) radar operation mode. HPMIMO adopts a phased-array mode inside the subarray and operates in the MIMO mode among subarrays. It contains the coherent processing gain of PAR and the MIMO waveform diversity gain of the radar. Hassanien et al. divided a transmitting array into a number of uniformly overlapping subarrays in 2010 to address the problem of subarray grouping for one-dimensional linear arrays. Furthermore, to design HPMIMO radar based on uniformly overlapping subarrays, they transmitted the signal to the target in the phased-array mode. Moreover, they transmitted mutually orthogonal signals between them in the MIMO radar mode [23]. In 2013, one study [24] proposed extending the HPMIMO radar operating mode to resolve the subarray grouping issue of 2D planar arrays. The main objective of the study was to divide the space into segments by generating beam patterns to be applied to the target tracking mode. For HPMIMO radar performance analysis, the modal function of HPMIMO radar was derived based on overlapping subarrays, and the mathematical properties of the ambiguity function were analyzed [25]. A joint transmit subarray grouping and beamforming design method was proposed by J.S. Huang et al. [26], where the transmit array was equally divided into a certain number of non-overlapping subarrays based on 2D HPMIMO radar. Subsequently, a joint optimization model was established for the subarray structure, transmitted beam power vector, and received beam power vector under the constraint of maximizing the output SINR. Finally, the model was solved in a circular, iterative manner. However, the method requires several iterations for algorithm optimization; therefore, it is less flexible and adaptable. In 2019, Tahcfulloh et al. divided the transmitting and receiving arrays into overlapping subarrays with the same number of array elements. Furthermore, they proposed an operating mode for FPMIMO radar [27] using a phased-array approach for signal transmission and reception in subarrays. In this mode, the receiver and transmitter were co-located, and various waveform combinations were transmitted using multiple subarrays to effectively improve the coherence and waveform diversity gains, along with exhibiting system flexibility. However, research on FPMIMO radars in terms of the air spatial filtering algorithm is rather limited and lacks robust algorithm designs.

This paper mainly studies the sparse optimization problem based on two-dimensional subarrays. Under the given array aperture, number of subarrays, and size of the subarrays, while satisfying the antenna aperture, eliminating grating lobes, and ensuring no overlap of subarrays, the optimization objective is to minimize the PSLL and the main lobe width as much as possible. By establishing an optimization mathematical model, the positions of the subarrays are optimized and solved. For multiple optimization results, the optimal solution is selected using the radar ambiguity function. Finally, a two-dimensional subarray-level antenna sparse optimization design applicable to phased array MIMO radar is achieved. This can improve the radar’s spatial resolution while suppressing grating lobes and high side lobes and significantly reduce the size of the subarrays in the array antenna, the hardware cost for data acquisition and processing, and energy consumption. This is of great significance for the development and application of radar technology. For the sparse optimization problem of array antennas, it is a nonlinear and nonconvex optimization problem. Usually, intelligent optimization algorithms are adopted, and the optimal solution is achieved through iterative looping to find the best solution, such as a GA, PSO, simulated annealing, or the ant colony algorithm. This study focuses on the problem of the optimal arrangement of sparse antenna arrays and proposes a new optimal design based on the GA–PSO hybrid optimization algorithm and ambiguity function in the FPMIMO radar operation mode. The proposed algorithm can adaptively adjust the number of optimization iterations according to the optimization results during optimization of the PSO algorithm and GA. This strongly ensures the global optimization performance of the algorithm and determines the subarray’s sparse optimization scheme by combining the ambiguity function graphs of the subarray’s sparse optimization results. This manuscript contains the following sections. Section 2 introduces the problem and algorithm model by first describing a sparse antenna array of 2D subarrays, then establishing an optimization model for array designing and a signal model for an array in FPMIMO mode, and finally deriving the ambiguity function of the array. Section 3 describes the implementation steps of the algorithm comprehensively. Section 4 presents a simulation test to verify the effectiveness of the algorithm. The conclusions of this study are given in Section 5.

## 2. Problem Description and Algorithm Model

### 2.1. Optimization Model for a Sparse Antenna Array of Two-Dimensional Subarrays

**Figure 1:**
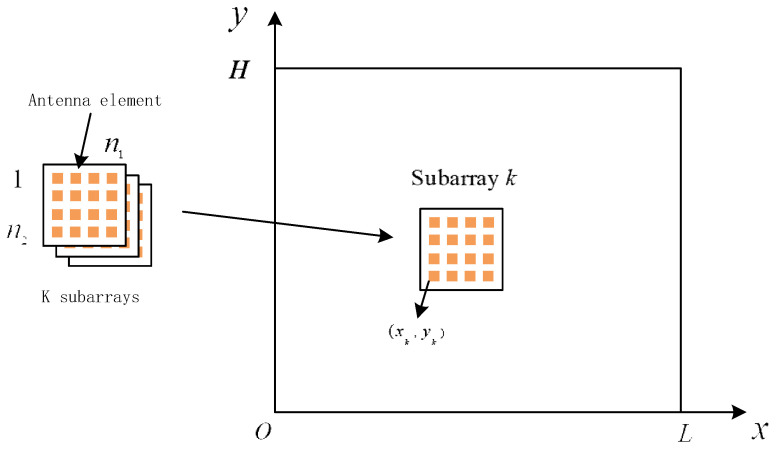
Schematic diagram of a sparse antenna array of two-dimensional subarrays.

As shown in Figure 1, the problem encountered by a sparse antenna array of 2D subarrays is the arrangement of K rectangular $n_{1}\times n_{2}$ subarrays on a plane xoy, with subarray elements being uniformly arranged at an interval of half the wavelength of the signal d. Moreover, subarrays are randomly arranged and constrained within the range of L×H, the elements of any two subarrays are nonoverlapping, and each subarray is uniquely identified using coordinates. These coordinates are the element in the first row and first column, i.e., $(x_{k},y_{k}),1\leq k\leq K$. Therefore, the coordinates of a subarray must satisfy the constraints $0\leq x_{k}\leq L_{m}$ and $0\leq y_{k}\leq H_{m}$, where $L_{m}\;=\;L\;-\;(n_{1}-1)\cdot d$ and $H_{m}\;=\;H\;-\;(n_{2}-1)\cdot d$. Conversely, to improve the spatial resolution of an array antenna, the array aperture must be expanded. To establish a fitness function, the minimization of the main lobe width can be set as another optimization objective. Thus, the array optimization problem is transformed into solving the position vector of N subarrays, and the optimization variables can be expressed as follows:

(1) $$d=\;{\left(\begin{array}{ccc} x_{1} & \ldots & x_{K}\\ y_{1} & \ldots & y_{K} \end{array}\right)}^{T}$$

where $(x_{k},y_{k}{)}^{T}$ is the position coordinates of the subarray k. In a sparse antenna array, because there are no overlapping elements between any two subarrays, the spacing between any two subarrays must satisfy any one of the minimum spacing constraints given in the following equation, neglecting the fact that the subarray spacing is less than half the wavelength d; in other words, we have

(2) $$\left|x_{k1}-x_{k2}\right|>(n_{1}-1)d,or\;\left|y_{k1}-y_{k2}\right|>(n_{2}-1)d$$

where k1 and k2 are any two different subarray numbers between 1 and N. Then, by minimizing the peak side lobe level (PSLL) and main lobe width of the array, the following optimization model can be established:

(3) $$\begin{array}{l} \underset{d}{\min}\;fitness=\underset{d}{\min}\;(\alpha_{1}\cdot PSLL+\alpha_{2}\cdot WB)\\ \mathrm{subject}\;\mathrm{to}\;0\leq x_{k}\leq L_{m},0\leq y_{k}\leq H_{m},\\ \;\;\;\;\;\;\left|x_{k1}-x_{k2}\right|>(n_{1}-1)d\;or\;\left|y_{k1}-y_{k2}\right|>(n_{2}-1)d,\\ \;\;\;\;\;\;1\leq k1,k2\leq K,k1\neq k2 \end{array}$$

where $\alpha_{1}$ and $\alpha_{2}$ are the regularization parameters, PSLL denotes the PSLL, and WB denotes the main lobe width. The optimization model shown in Equation (3) is a nonlinear, nonconvex optimization problem that cannot be solved directly using the existing optimization software and is usually solved iteratively by employing an intelligent optimization algorithm, where the optimization variable is the position vector of d.

To ensure the robustness of the optimization results, a relaxation function is introduced. By setting the expected values of the PSLL $PSLL_{d}$ and the main lobe width $WB_{d}$, when the error of either item exceeds a certain limit of the expected value, the fitness function value will increase exponentially. This can effectively avoid unfavorable results, where the PSLL is extremely small while the main lobe width is relatively large or the main lobe width is extremely small while the PSLL is relatively large. This ensures that the optimization results of the proposed algorithm accurately fall within the predetermined interval. Thus, under the condition that the optimization results simultaneously meet the expected values of the PSLL and the main lobe width, the optimal solution for the system design can be automatically found. Therefore, a new fitness function can be developed as follows:

(4) $$fitness=\;\frac{\alpha_{1}PSLL}{\left|{PSLL}_{d}\right|}+\;\frac{\alpha_{2}{WB}_{\max}}{{WB}_{d}}+h_{m}+\;h_{w}$$

where $h_{m}=e^{\beta_{1}\cdot \max(0,\;PSLL-PSLL_{d}-Pe)}$, $h_{w}=e^{\beta_{2}\cdot \max(0,\;WB_{\max}-WB_{d}-W_{e})}$, $\left|\cdot \right|$ denotes absolute values, $h_{m}$ is the relaxation function of the PSLL error, Pe is the upper limit of the allowed error for the PSLL, $h_{w}$ is the relaxation function of the main lobe width error, and $WB_{max}$ is the maximum width of the main lobe in the beam pattern. Because the main lobe of a 2D array is a cone, the maximum value of the main lobe width is used as the corresponding main lobe width for the subarray optimization scheme. $W_{e}$ is the upper limit of the allowed error for the main lobe width, and $\beta_{1}$ and $\beta_{2}$ are the positive amplification factors. The upper error limits for Pe and $W_{e}$ in Equation (4) are essentially introduced as redundancy values to prevent the expected PSLL $PSLL_{d}$ and main lobe width $WB_{d}$ from being set to unreasonable values, which may lead to oscillations in the optimization results. They can reveal alternatives and help improve the robustness of the algorithm.

**Figure 2:**
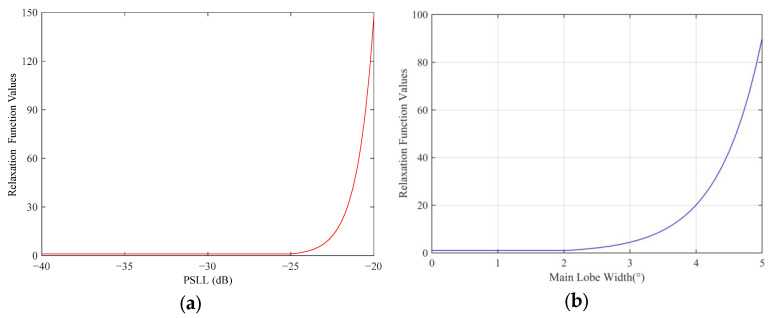
Relaxation function graph. (**a**) Relaxation function curve of the PSLL. (**b**) Relaxation function curve of the main lobe width.

**Figure 3:**
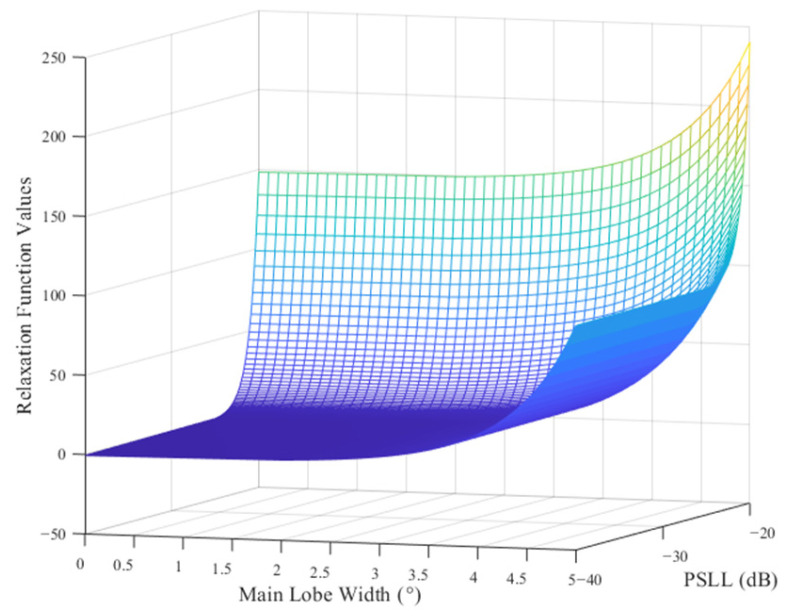
Adaptation function surface.

The simulation results for the relaxation function of the PSLL error $h_{m}$ are shown in Figure 2a, along with the expected PSLL value $PSLL_{d}\;=\;-30\;\mathrm{dB}$, upper error limit Pe = 5 dB, and amplification factor $\beta_{1}\;=\;1$. Furthermore, the simulation results for the relaxation function of the main lobe width error $h_{w}$ are shown in Figure 2b, along with the expected main lobe width $WB_{d}\;=\;1.5^{\circ }$, upper error limit $W_{e}\;=\;0.5^{\circ }$, and amplification factor $\beta_{2}\;=\;1.5$. These simulation results show that by setting the upper error limit, the minimum interval of the relaxation function is widened, which is more conducive to the search for a near-optimal grouping scheme, providing more alternatives to the intelligent algorithm for the optimal search process and improving the search probability of the algorithm to find the optimal subarray grouping scheme. The surface of the fitness function is shown in Figure 3. When the PSLL or main lobe width is within predefined error ranges, the fitness function value increases slowly, and when any of them exceeds the predefined error range, the value of the fitness function increases sharply, which enables rapidly eliminating the inappropriate subarray grouping scheme and helps the optimization algorithm in identifying a reduced range before searching for the optimal value.

### 2.2. FPMIMO Signal Model for a Sparse Antenna Array of 2D Subarrays

In FPMIMO mode, the receiver and transmitter are co-located in an array. The array antenna is composed of 2D sparse subarrays. Subarrays function internally in the phased-array mode for transmission and reception and function in MIMO mode among them. Therefore, FPMIMO radar not only has the coherent processing gain for signal transmission and reception but also has waveform diversity gain among the subarrays, which is a new radar operation mode with the deep integration of PAR and MIMO radars.

Suppose an array antenna comprises K subarrays, and each subarray is rectangular with elements at an interval of half the wavelength d, and the position of each subarray is shown in Equation (1). Each subarray transmits a signal with a unit energy but different waveforms. Then, the transmitting waveform vector can be defined by

(5) $$s\left(t\right)≜\;[s_{0}(t),s_{1}(t),..\;.,s_{K-1}(t){]}^{T}$$

Assuming that the radar divides the transmit power of all subarrays by an equal power, the complex envelope vector of the transmit signal for the k subarray can be expressed as follows:

(6) $$\psi_{k}\left(t\right)\;=\;\sqrt{\frac{P}{K}}s_{k}(t)w_{k}^{*}$$

where P is the total power of the radar transmit signal, $w_{k}$ is the weight vector when the k subarray transmits the signal, √ denotes the open radical operation, and $(\cdot {)}^{*}$ represents conjugation. Then, the total emitted signal from the array of 2D subarrays in the $\left(\varphi_{0},\theta_{0}\right)$ direction can be written as follows:

(7) $$s\left(t,\varphi_{0},\theta_{0}\right)\;=\;\sum_{k\;=\;0}^{K-1}\psi_{k}^{T}(t)a_{k}(\varphi_{0},\theta_{0})e^{-j2\pi f_{c}\frac{\left(x_{k}\cos\varphi_{0}\sin\theta_{0}+y_{k}\sin\varphi_{0}\sin\theta_{0}\right)}{c}}$$

where $\varphi_{0}$ is the azimuth angle, $\theta_{0}$ is the elevation angle, $(x_{k},y_{k}{)}^{T}$ is the position of the reference array element of the k subarray, $f_{c}$ is the transmit signal carrier frequency, and $a_{k}(\varphi ,\theta )$ is the transmit signal steering vector of the k subarray, which can be expressed by

(8) $$a_{k}\left(\varphi_{0},\theta_{0}\right)=\mathrm{vec}(a_{1}(\varphi_{0},\theta_{0})a_{2}^{T}(\varphi_{0},\theta_{0}))$$

where vec (⋅) denotes rearranging the elements of a matrix into a vector:

(9) $$a_{1}\left(\varphi_{0},\theta_{0}\right)\;=\;[1,e^{-j2\pi f_{c}\frac{d\sin\varphi_{0}\sin\theta_{0}}{c}},...,e^{-j2\pi f_{c}\frac{(n_{1}-1)d\sin\varphi_{0}\sin\theta_{0}}{c}}{]}^{T}$$

(10) $$a_{2}\left(\varphi_{0},\theta_{0}\right)=[1,e^{-j2\pi f_{c}\frac{d\cos\varphi_{0}\sin\theta_{0}}{c}},...,e^{-j2\pi f_{c}\frac{(n_{2}-1)d\cos\varphi_{0}\sin\theta_{0}}{c}}{]}^{T}$$

where $a_{1}(\varphi_{0},\theta_{0})$ is the steering vector of the first column and $a_{2}(\varphi_{0},\theta_{0})$ is the steering vector of the first row of the subarray. After neglecting the target reflection coefficient, the received signal vector of the array is obtained, as reported in [25]:

(11) $$\begin{array}{l} x(t,\tau ,f_{d},\varphi_{0},\theta_{0})\\ \;\;\;\;=\sqrt{P/K}b(\varphi_{0},\theta_{0})\sum_{k=0}^{K-1}w_{k}^{H}a_{k}(\varphi_{0},\theta_{0})e^{-j2\pi f_{c}\left(x_{k}\cos\varphi_{0}\sin\theta_{0}+y_{k}\sin\varphi_{0}\sin\theta_{0}\right)/c}s_{k}(t-\tau )e^{j2\pi f_{d}t} \end{array}$$

where $b(\varphi_{0},\theta_{0})$ is the array receive steering vector, τ is the signal’s round-trip delay between the target and antenna, and $f_{d}$ is the Doppler frequency due to the target motion. According to Equation (5), the above equation can be further written as follows:

(12) $$x\left(t,\tau ,f_{d},\varphi_{0},\theta_{0}\right)\;=\;\sqrt{\frac{P}{K}}b(\varphi_{0},\theta_{0})[c(\varphi_{0},\theta_{0})⊙d(\varphi_{0},\theta_{0}){]}^{T}s(t-\tau )e^{j2\pi f_{d}t}$$

where

(13) $$c\left(\varphi_{0},\theta_{0}\right)\;≜\;[w_{0}^{H}a_{0}(\varphi_{0},\theta_{0}),w_{1}^{H}a_{1}(\varphi_{0},\theta_{0}),...,w_{K-1}^{H}a_{K-1}(\varphi_{0},\theta_{0}){]}^{T}$$

(14) $$d(\varphi_{0},\theta_{0})≜{\left[1,e^{-j2\pi f_{c}\left(x_{2}\cos\varphi_{0}\sin\theta_{0}+y_{2}\sin\varphi_{0}\sin\theta_{0}\right)/c},\ldots ,e^{-j2\pi f_{c}\left(x_{K-1}\cos\varphi_{0}\sin\theta_{0}+y_{K-1}\sin\varphi_{0}\sin\theta_{0}\right)/c}\right]}^{T}$$

where $c(\varphi_{0},\theta_{0})$ is the coherent processing gain vector of the signal emission and $d(\varphi_{0},\theta_{0})$ is the waveform diversity vector.

For FPMIMO radar, its signal is still received in the phased-array mode of subarrays, and the subarrays function in MIMO mode among them. Therefore, the steering vector of the received signal is obtained as follows:

(15) $$b\left(\varphi_{0},\theta_{0}\right)\;=\;g(\varphi_{0},\theta_{0})⊙d(\varphi_{0},\theta_{0})$$

where

(16) $$g(\varphi_{0},\theta_{0})≜[u_{0}^{H}a_{0}(\varphi_{0},\theta_{0}),u_{1}^{H}a_{1}(\varphi_{0},\theta_{0}),...,u_{K-1}^{H}a_{K-1}(\varphi_{0},\theta_{0}){]}^{T}$$

where $g(\varphi_{0},\theta_{0})$ is the received coherent processing gain vector and $u_{k}$ is the signal reception power vector inside the subarray.

Based on the above analysis, the signal received from an FPMIMO radar with a sparse antenna array of 2D subarrays can be expressed as follows:

(17) $$x\left(t,\tau ,f_{d},\varphi_{0},\theta_{0}\right)\;=\;\;\sqrt{\frac{P}{K}}[g(\varphi_{0},\theta_{0})⊙d(\varphi_{0},\theta_{0})]\cdot [c(\varphi_{0},\theta_{0})⊙d(\varphi_{0},\theta_{0}){]}^{T}s(t-\tau )e^{j2\pi f_{d}t}$$

The above equation is a function of the transmit coherent processing gain vector of the array $c(\varphi_{0},\theta_{0})$, waveform diversity vector $d(\varphi_{0},\theta_{0})$, and received coherent processing gain vector $g(\varphi_{0},\theta_{0})$. Because the system uses the phased-array method of subarrays for signal transmission and reception and the subarrays function in MIMO radar mode among them, the steering vectors inside the subarrays and waveform diversity vectors are the same for signal transmission and reception.

### 2.3. Radiation Pattern of a Sparse Antenna Array of 2D Subarrays

The FPMIMO radar system based on a sparse antenna array of 2D subarrays has a signal transmit coherent processing gain vector, a waveform diversity vector, and a receive coherent processing gain vector. The radiation patterns of the transmit coherent processing gain and waveform diversity are determined by the transmitter end of the radar system, and its receiver end still has the receive coherent processing gain and waveform diversity vectors as the receiver and transmitter are co-located. Thus, the radiation pattern of the FPMIMO radar array based on a sparse antenna array of 2D subarrays comprises the radiation patterns of the transmit coherent processing gain and waveform diversity and a receive radiation pattern [28].

From the analysis in the previous section, the radiation pattern of the transmit coherent processing gain is given by

(18) $$F_{tra}\left(\varphi ,\theta \right)=\frac{{\left|c^{H}(\varphi_{0},\theta_{0})c(\varphi ,\theta )\right|}^{2}}{{\left\|c^{H}(\varphi_{0},\theta_{0})\right\|}^{4}}$$

The radiation pattern of the waveform diversity is

(19) $$F_{div}\left(\varphi ,\theta \right)=\frac{{\left|d^{H}(\varphi_{0},\theta_{0})d(\varphi ,\theta )\right|}^{2}}{{\left\|d(\varphi_{0},\theta_{0})\right\|}^{4}}$$

The receive radiation pattern comprises the radiation patterns of the coherent processing gain and waveform diversity; in other words, we have

(20) $$\begin{array}{c} F_{rec}(\varphi ,\theta )=\frac{{\left|b^{H}(\varphi_{0},\theta_{0})b(\varphi ,\theta )\right|}^{2}}{{\left\|b(\varphi_{0},\theta_{0})\right\|}^{4}}\\ \;\;\;\;\;\;\;\;\;=\frac{{\left|g^{H}(\varphi_{0},\theta_{0})g(\varphi ,\theta )\right|}^{2}}{{\left\|g(\varphi_{0},\theta_{0})\right\|}^{4}}\cdot \frac{{\left|d^{H}(\varphi_{0},\theta_{0})d(\varphi ,\theta )\right|}^{2}}{{\left\|d(\varphi_{0},\theta_{0})\right\|}^{4}} \end{array}$$

where $\left\|\cdot \right\|$ denotes the Euclidean norm. Based on the above analysis, the radiation pattern of the coherent processing gain and waveform diversity as well as the receive radiation pattern of the array transmit signal can be obtained. Additionally, the transmit radiation pattern of a sparse antenna array of 2D subarrays can be expressed as follows:

(21) $$\begin{array}{c} F_{transmit}=F_{tra}(\varphi ,\theta )\cdot F_{div}(\varphi ,\theta )\\ \;\;\;\;\;\;\;=\frac{{\left|c^{H}(\varphi_{0},\theta_{0})c(\varphi ,\theta )\right|}^{2}}{{\left\|c^{H}(\varphi_{0},\theta_{0})\right\|}^{4}}\cdot \frac{{\left|d^{H}(\varphi_{0},\theta_{0})d(\varphi ,\theta )\right|}^{2}}{{\left\|d(\varphi_{0},\theta_{0})\right\|}^{4}} \end{array}$$

Accordingly, the total radiation pattern of an FPMIMO radar based on a sparse antenna array of 2D subarrays can be expressed by

(22) $$\begin{array}{c} F(\varphi ,\theta )=F_{tra}(\varphi ,\theta )\cdot F_{div}(\varphi ,\theta )\cdot F_{rec}(\varphi ,\theta )\\ \;\;\;\;\;\;\;=\frac{{\left|c^{H}(\varphi_{0},\theta_{0})c(\varphi ,\theta )\right|}^{2}}{{\left\|c^{H}(\varphi_{0},\theta_{0})\right\|}^{4}}\cdot \frac{{\left|d^{H}(\varphi_{0},\theta_{0})d(\varphi ,\theta )\right|}^{4}}{{\left\|d(\varphi_{0},\theta_{0})\right\|}^{8}}\cdot \frac{{\left|g^{H}(\varphi_{0},\theta_{0})g(\varphi ,\theta )\right|}^{2}}{{\left\|g(\varphi_{0},\theta_{0})\right\|}^{4}} \end{array}$$

### 2.4. Ambiguity Function of the Sparse Antenna Array of 2D Subarrays

At the receiving end, assuming that the parameters used by matched filtering (MF) for signal processing are $\tau_{r}$, $f_{r}$, $\varphi_{r}$, and $\theta_{r}$, whose definition is p(φ,θ)≜c(φ,θ)⊙d(φ,θ). Using Equations (15) and (17), the MF output can be further expressed by

(23) $$\begin{array}{l} \int_{-\infty }^{+\infty }x^{H}(t,\tau_{r},f_{r},\varphi_{r},\theta_{r})x(t,\tau ,f_{d},\varphi_{0},\theta_{0})dt\\ =b^{H}(\varphi_{r},\theta_{r})b(\varphi_{0},\theta_{0})\int_{-\infty }^{+\infty }\frac{P}{K}s^{H}(t-\tau_{r}){\left[p(\varphi_{r},\theta_{r})\right]}^{*}{\left[p(\varphi_{0},\theta_{0})\right]}^{T}s(t-\tau )e^{j2\pi (f_{d}-f_{r})t}dt \end{array}$$

According to the above equation, the first term on the right-hand side of the equation indicates spatial data processing at the receiver side, and the sparse antenna array of 2D subarrays will affect the MF processing results. The second integral term reflects the spatial, Doppler, and distance resolutions of the radar. Therefore, the generalized ambiguity function of an FPMIMO radar based on an array of 2D subarrays can be defined by

(24) $$\begin{array}{l} \gamma (\tau ,f_{d},\varphi_{0},\theta_{0},\tau_{r},f_{r},\varphi_{r},\theta_{r})\\ =\frac{P}{K}b^{H}(\varphi_{r},\theta_{r})b(\varphi_{0},\theta_{0})\int_{-\infty }^{+\infty }s^{H}(t-\tau_{r}){\left[p(\varphi_{r},\theta_{r})\right]}^{*}{\left[p(\varphi_{0},\theta_{0})\right]}^{T}s(t-\tau )e^{j2\pi (f_{d}-f_{r})t}dt\\ =\frac{P}{K}[b^{H}(\varphi_{r},\theta_{r})b(\varphi_{0},\theta_{0})]\cdot [p(\varphi_{0},\theta_{0}){]}^{T}\int_{-\infty }^{+\infty }s(t-\tau )s^{H}(t-\tau_{r})e^{j2\pi (f_{d}-f_{r})t}dt\cdot [p(\varphi_{r},\theta_{r}){]}^{*} \end{array}$$

With the delay error being defined as $\tilde{\tau }\;=\;\tau \;-\;\tau_{r}$ and multifrequency error being ${\tilde{f}}_{d}\;=\;f_{d}\;-\;f_{r}$, $\tilde{t}\;=\;t\;-\;\tau$, the above equation can be further expressed as follows:

(25) $$\begin{array}{l} \gamma (\tilde{\tau },{\tilde{f}}_{d},\varphi_{0},\theta_{0},f_{r},\varphi_{r},\theta_{r})\\ =\frac{P}{K}[b^{H}(\varphi_{r},\theta_{r})b(\varphi_{0},\theta_{0})][p(\varphi_{0},\theta_{0}){]}^{T}\int_{-\infty }^{+\infty }s(\tilde{t})s^{H}(\tilde{t}+\tilde{\tau })e^{j2\pi {\tilde{f}}_{d}\tilde{t}}d\tilde{t}\cdot [p(\varphi_{r},\theta_{r}){]}^{*} \end{array}$$

According to Equation (5), the transmit signal vector of the radar is composed of K mutually orthogonal linear frequency modulation (LFM) signals, where the transmitting signal of the k subarray can be expressed by

(26) $$s_{k}(t)=\mathrm{rect}(\frac{t}{T_{p}})\exp[j2\pi (f_{0}t+\frac{1}{2}\mu t^{2}+kf_{m}t)]$$

where μ is the tuning frequency, $f_{0}$ is the carrier frequency, and $f_{m}$ is the frequency interval between LFM signals. According to Equations (25) and (26), the mutual ambiguity function can be defined by

(27) $$\chi_{k_{1},k_{2}}(\tilde{\tau },{\tilde{f}}_{d})=\int_{-\infty }^{+\infty }s_{k_{1}}(t)s_{k_{2}}^{*}(t+\tilde{\tau })\exp(j2\pi {\tilde{f}}_{d}t)dt$$

where $k_{1}$ and $k_{2}$ are any integers from 0 to N − 1.

There are K LFM signals forming the vector $s\left(t\right)={\left[s_{0}\left(t\right),s_{1}\left(t\right),\ldots ,s_{K-1}\left(t\right)\right]}^{T}$, where the $k_{1}$ and $k_{2}$ transmit signals can be expressed as follows:

(28) $$s_{k_{1}}(t)=\mathrm{rect}(\frac{t}{T_{p}})\exp[j2\pi (f_{0}t+\frac{1}{2}\mu t^{2}+k_{1}f_{m}t)]$$

(29) $$s_{k_{2}}(t)=\mathrm{rect}(\frac{t}{T_{p}})\exp[j2\pi (f_{0}t+\frac{1}{2}\mu t^{2}+k_{2}f_{m}t)]$$

According to the definition of the mutual ambiguity function in Equation (27), for the delay error τ>0, we have

(30) $$\begin{array}{l} \chi_{k_{1},k_{2}}(\tau ,f_{d})\\ =\int_{-\frac{T_{p}}{2}}^{\frac{T_{p}}{2}-\tau }\exp(j2\pi (f_{0}t+\frac{1}{2}\mu t^{2}+k_{1}f_{m}t))\exp(-j2\pi (f_{0}(t+\tau )+\frac{1}{2}\mu (t+\tau {)}^{2}+k_{2}f_{m}(t+\tau )))e^{j2\pi f_{d}t}dt\\ =\exp(-j2\pi (f_{0}\tau +\frac{1}{2}\mu \tau^{2}+k_{2}f_{m}\tau ))\int_{-\frac{T_{p}}{2}}^{\frac{T_{p}}{2}-\tau }\exp(j2\pi ((k_{1}-k_{2})f_{m}t-\mu \tau t+f_{d}t))dt \end{array}$$

In the above equation, let $\varphi \;=\left(k_{1}-k_{2}\right)f_{m}-\mu \tau \;+f_{d}$ and $\psi \left(\tau \right)=\exp(-j2\pi \;(f_{0}\tau \;+\frac{1}{2}\mu \tau^{2}+{\;k}_{2}f_{m}\tau ))$, and we have

(31) $$\begin{array}{c} \chi_{k_{1},k_{2}}(\tau ,f_{d})=\psi (\tau )\int_{-\frac{T_{p}}{2}}^{\frac{T_{p}}{2}-\tau }\exp(j2\pi \varphi t)dt\\ \;\;\;\;\;\;\;\;\;\;\;=\frac{\psi (\tau )}{j2\pi \varphi }[\exp(j\pi \varphi (T_{p}-2\tau ))-\exp(-j\pi \varphi T_{p})]\\ \;\;\;\;\;\;\;\;\;\;\;=\frac{\psi (\tau )}{j2\pi \varphi }[\exp(-j2\pi \varphi \tau )(\cos(\pi \varphi T_{p})+j\sin(\pi \varphi T_{p}))-\cos(\pi \varphi T_{p})+j\sin(\pi \varphi T_{p})]\\ \;\;\;\;\;\;\;\;\;\;\;=\frac{\psi (\tau )}{\pi \varphi }\exp(-j\pi \varphi \tau )[\sin(\pi \varphi T_{p})\cos(\pi \varphi \tau )-\cos(\pi \varphi T_{p})\sin(\pi \varphi \tau )]\\ \;\;\;\;\;\;\;\;\;\;\;=\frac{\psi (\tau )}{\pi \varphi }\exp(-j\pi \varphi \tau )\sin(\pi \varphi (T_{p}-\tau )) \end{array}$$

By substituting φ and Ψ(τ) we have

(32) $$\begin{array}{l} \chi_{k_{1},k_{2}}(\tau ,f_{d})\\ =\exp(-j2\pi (f_{0}\tau +\frac{1}{2}\mu \tau^{2}+k_{2}f_{m}\tau ))\exp(-j\pi ((k_{1}-k_{2})f_{m}-\mu \tau +f_{d})\tau )\\ \times \frac{\sin(\pi ((k_{1}-k_{2})f_{m}-\mu \tau +f_{d})(T_{p}-\tau ))}{\pi ((k_{1}-k_{2})f_{m}-\mu \tau +f_{d})}\\ =(T_{p}-\tau )\exp(-j2\pi (f_{0}\tau +\frac{1}{2}\mu \tau^{2}+k_{2}f_{m}\tau ))\exp(-j\pi ((k_{1}-k_{2})f_{m}-\mu \tau +f_{d})\tau )\\ \times \frac{\sin(\pi ((k_{1}-k_{2})f_{m}-\mu \tau +f_{d})(T_{p}-\tau ))}{\pi ((k_{1}-k_{2})f_{m}-\mu \tau +f_{d})(T_{p}-\tau )}\\ =T_{p}\exp(-j2\pi (f_{0}\tau +\frac{1}{2}\mu \tau^{2}+k_{2}f_{m}\tau ))\exp(-j\pi ((k_{1}-k_{2})f_{m}-\mu \tau +f_{d})\tau )\\ \times \sin c(\pi ((k_{1}-k_{2})f_{m}-\mu \tau +f_{d})(T_{p}-\tau ))\cdot (1-\frac{\tau }{T_{p}}) \end{array}$$

For τ<0, the mutual ambiguity function $\chi_{k_{1},k_{2}}(\tau ,f_{d})$ can be expressed by

(33) $$\begin{array}{l} \chi_{k_{1},k_{2}}(\tau ,f_{d})\\ =\int_{-\frac{T_{p}}{2}-\tau }^{\frac{T_{p}}{2}}\exp(j2\pi (f_{0}t+\frac{1}{2}\mu t^{2}+k_{1}f_{m}t))\exp(-j2\pi (f_{0}(t+\tau )+\frac{1}{2}\mu (t+\tau {)}^{2}+k_{2}f_{m}(t+\tau )))e^{j2\pi f_{d}t}dt\\ =\exp(-j2\pi (f_{0}\tau +\frac{1}{2}\mu \tau^{2}+k_{2}f_{m}\tau ))\int_{-\frac{T_{p}}{2}-\tau }^{\frac{T_{p}}{2}}\exp(j2\pi ((k_{1}-k_{2})f_{m}-\mu \tau +f_{d}))t)dt \end{array}$$

In the above equation, let $\varphi =(k_{1}-k_{2})f_{m}-\mu \tau +f_{d}$ and $\psi (\tau )=\exp(-j2\pi (f_{0}\tau +\frac{1}{2}\mu \tau^{2}+k_{2}f_{m}\tau ))$. Then, we have

(34) $$\begin{array}{c} \chi_{k_{1},k_{2}}(\tau ,f_{d})=\psi (\tau )\int_{-\frac{T_{p}}{2}-\tau }^{\frac{T_{p}}{2}}\exp(j2\pi \varphi t)dt\\ \;\;\;\;\;\;\;\;\;\;=\frac{\psi (\tau )}{j2\pi \varphi }(\exp(j\pi \varphi T_{p})-\exp(j\pi \varphi T_{p}-j2\pi \varphi \tau ))\\ \;\;\;\;\;\;\;\;\;\;=\frac{\psi (\tau )}{j2\pi \varphi }(\cos(\pi \varphi T_{p})+j\sin(\pi \varphi T_{p})-\exp(-j2\pi \varphi \tau )(\cos(\pi \varphi T_{p})-j\sin(\pi \varphi T_{p})))\\ \;\;\;\;\;\;\;\;\;\;=\frac{\psi (\tau )}{\pi \varphi }\exp(-j\pi \varphi \tau )\sin(\pi \varphi (T_{p}+\tau )) \end{array}$$

By substituting φ and Ψ(τ), we have

(35) $$\begin{array}{l} \chi_{k_{1},k_{2}}(\tau ,f_{d})\\ =\exp(-j2\pi (f_{0}\tau +\frac{1}{2}\mu \tau^{2}+k_{2}f_{m}\tau ))\exp(-j\pi ((k_{1}-k_{2})f_{m}-\mu \tau +f_{d})\tau )\\ \times \frac{\sin(\pi ((k_{1}-k_{2})f_{m}-\mu \tau +f_{d})(T_{p}+\tau ))}{\pi ((k_{1}-k_{2})f_{m}-\mu \tau +f_{d})}\\ =T_{p}\exp(-j2\pi (f_{0}\tau +\frac{1}{2}\mu \tau^{2}+k_{2}f_{m}\tau ))\exp(-j\pi ((k_{1}-k_{2})f_{m}-\mu \tau +f_{d})\tau )\\ \times \sin c(\pi ((k_{1}-k_{2})f_{m}-\mu \tau +f_{d})(T_{p}+\tau ))\cdot (1+\frac{\tau }{T_{p}}) \end{array}$$

By combining Equations (32) and (35), we have the following mutual ambiguity function for the LFM signal:

(36) $$\begin{array}{c} \chi_{k_{1},k_{2}}(\tilde{\tau },{\tilde{f}}_{d})=T_{p}\exp\{-j2\pi (f_{0}\tilde{\tau }+\frac{1}{2}\mu {\tilde{\tau }}^{2}+k_{2}f_{m}\tilde{\tau })-j\pi [(k_{1}-k_{2})f_{m}-\mu \tilde{\tau }+{\tilde{f}}_{d}]\tilde{\tau }\}\\ \;\;\;\;\;\;\;\;\;\;\;\times \frac{\sin\{\pi [(k_{1}-k_{2})f_{m}-\mu \tau +f_{d}](T-\left|\tilde{\tau }\right|)\}}{\pi [(k_{1}-k_{2})f_{m}-\mu \tau +f_{d}](T-\left|\tilde{\tau }\right|)}(1-\frac{\left|\tilde{\tau }\right|}{T_{p}})\\ \;\;\;\;\;\;\;\;\;\;\;=T_{p}\exp\{-j2\pi (f_{0}\tilde{\tau }+\frac{1}{2}\mu {\tilde{\tau }}^{2}+k_{2}f_{m}\tilde{\tau })-j\pi [(k_{1}-k_{2})f_{m}-\mu \tilde{\tau }+{\tilde{f}}_{d}]\tilde{\tau }\}\\ \;\;\;\;\;\;\;\;\;\;\;\times \sin c\{\pi [(k_{1}-k_{2})f_{m}-\mu \tau +{\tilde{f}}_{d}](T_{p}-\left|\tilde{\tau }\right|)\}\cdot (1-\frac{\left|\tilde{\tau }\right|}{T_{p}}) \end{array}$$

Moreover, from Equations (25) and (36), we can further express the generalized ambiguity function as

(37) $$\begin{array}{c} \gamma (\tilde{\tau },{\tilde{f}}_{d},\varphi_{0},\theta_{0},f_{r},\varphi_{r},\theta_{r})\\ \;\;\;\;\;\;\;\;\;=\frac{P}{K}[b^{H}(\varphi_{r},\theta_{r})b(\varphi_{0},\theta_{0})]\\ \;\;\;\;\;\;\;\;\;\times \sum_{k_{1}=0}^{K-1}\sum_{k_{2}=0}^{K-1}\begin{array}{l} w_{k_{1}}^{H}a_{k_{1}}(\varphi_{0},\theta_{0})e^{-j2\pi f_{c}\left(x_{k_{1}}\cos\varphi_{0}\sin\theta_{0}+y_{k_{1}}\sin\varphi_{0}\sin\theta_{0}\right)/c}\cdot \chi_{k_{1},k_{2}}(\tilde{\tau },{\tilde{f}}_{d})\\ \times {\left(w_{k_{2}}^{H}a_{k_{2}}(\varphi_{r},\theta_{r})e^{-j2\pi f_{c}\left(x_{k_{2}}\cos\varphi_{r}\sin\theta_{r}+y_{k_{2}}\sin\varphi_{r}\sin\theta_{r}\right)/c}\right)}^{*} \end{array} \end{array}$$

If the conventional beamforming method is used inside each subarray, and the beam is pointed in the $\left(\varphi_{0},\theta_{0}\right)$ direction, then the signal transmit and receive power vectors inside the subarray are

(38) $$w_{k}=u_{k}=a_{k}(\varphi_{0},\theta_{0}),\;\;k=0,1,\ldots ,N-1$$

Then, we can also have

(39) $$\begin{array}{l} w_{k}^{H}a_{k}(\varphi_{0},\theta_{0})=n_{1}n_{2},w_{k}^{H}a_{k}(\varphi_{r},\theta_{r})=\mathrm{sum}(a_{k}(\varphi_{r}-\varphi_{0},\theta_{r}-\theta_{0}))\\ u_{k}^{H}a_{k}(\varphi_{0},\theta_{0})=n_{1}n_{2},\;\;u_{k}^{H}a_{k}(\varphi_{r},\theta_{r})=\mathrm{sum}(a_{k}(\varphi_{r}-\varphi_{0},\theta_{r}-\theta_{0})) \end{array}$$

where sum (⋅) denotes the sum of the elements of the vector. Therefore, the generalized ambiguity function of the FPMIMO radar based on a sparse antenna array of 2D subarrays is

(40) $$\begin{array}{l} \gamma (\tilde{\tau },{\tilde{f}}_{d},\varphi_{0},\theta_{0},f_{r},\varphi_{r},\theta_{r})\\ =\frac{P(n_{1}n_{2}{)}^{2}}{K}{\left|\mathrm{sum}(a_{k}(\varphi_{r}-\varphi_{0},\theta_{r}-\theta_{0}))\right|}^{2}\mathrm{sum}(d(\varphi_{0}-\varphi_{r},\theta_{0}-\theta_{r}))\\ \times \sum_{k_{1}=0}^{K-1}\sum_{k_{2}=0}^{K-1}e^{-j2\pi f_{c}\left(x_{k_{1}}\cos\varphi_{0}\sin\theta_{0}-x_{k_{2}}\cos\varphi_{r}\sin\theta_{r}+y_{k_{1}}\sin\varphi_{0}\sin\theta_{0}-y_{k_{2}}\sin\varphi_{r}\sin\theta_{r}\right)/c}\cdot \chi_{k_{1},k_{2}}(\tilde{\tau },{\tilde{f}}_{d}) \end{array}$$

According to the above equation, the generalized ambiguity function is closely related to the positions of the sparse subarrays, their internal structures and sizes, and the mutual ambiguity function of the transmitted signal. Hence, the generalized ambiguity function can be used for the selection of the optimal array scheme.

## 3. Algorithm Implementation Steps

Based on the above analysis, the basic idea of the algorithm introduced in this section is to calculate the main lobe width and PSLL for the subarray grouping scheme according to the transmit radiation pattern described in Equation (21), determine the fitness value described in Equation (4), and provide a basis for evaluating the array scheme. A position vector of a sparse antenna array of subarrays with optimal fitness can be obtained through the circular iterative optimization of the GA–PSO hybrid algorithm. The position vector is then combined with the ambiguity function of the FPMIMO radar based on a sparse antenna array of 2D subarrays obtained in Equation (40) to determine the optimal sparse antenna array scheme.

In the optimized sparse antenna array of 2D subarrays, an improved GA–PSO hybrid algorithm is proposed for optimizing the array design of 2D subarrays. This method integrates the global optimization capability of the GA algorithm and fast local optimization performance of the PSO algorithm such that the chromosomes in the GA algorithm correspond to particles in the PSO algorithm. According to the objective function of the problem, a fitness function is established based on the objective parameters. In the GA algorithm, the diversity of species is enhanced via cyclic evaluation, selection, crossover, and mutation operations on a species comprising several solutions to avoid local optimal solutions in order to obtain a group of individuals with the best fitness as the optimal solution for the problem as a whole. In the PSO algorithm, the chromosomes in the GA algorithm are given an initial velocity, and the local optimal solution is solved based on the optimal solution set using the fast optimization capability of the PSO algorithm. The hybrid algorithm combines the merits of the GA and PSO algorithms, reduces its dependence on the initial specifics, achieves adaptive convergence, and enhances the global optimization ability of the GA algorithm with the fast local optimization performance of the PSO algorithm.

**Figure 4:**
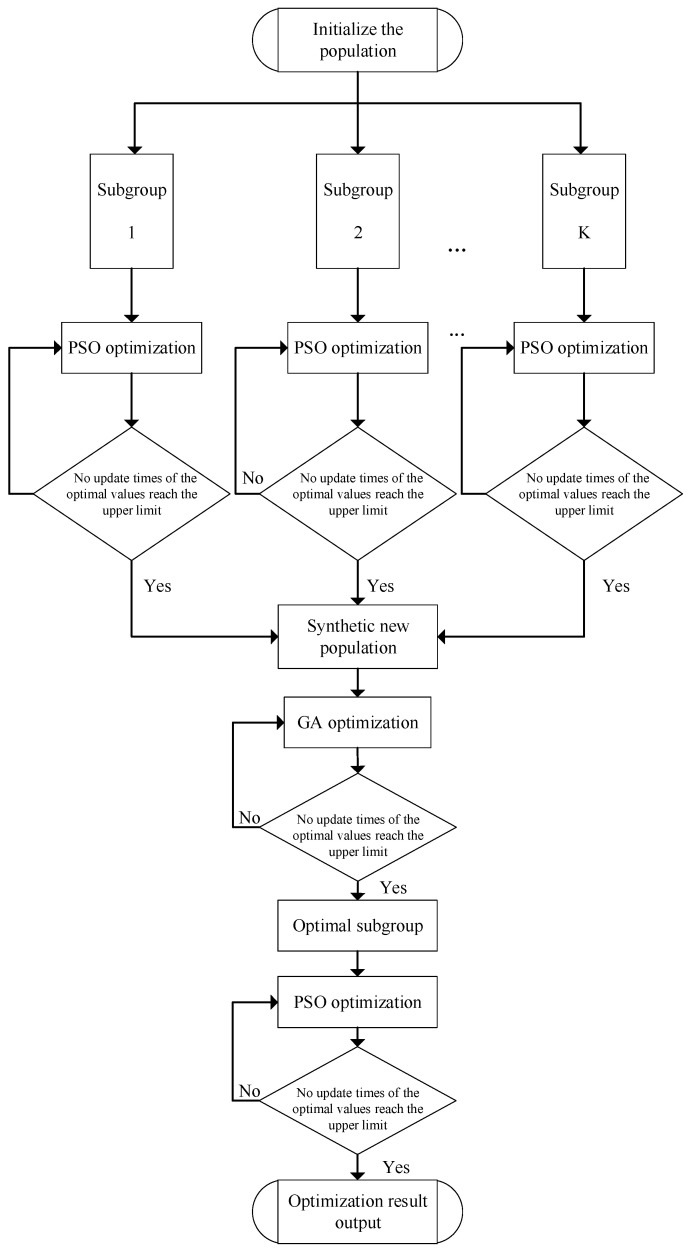
Flow chart of the GA-PSO hybrid algorithm.

The flowchart of the GA–PSO hybrid algorithm is shown in Figure 4, and the specific steps of the algorithm are described as follows.

Step 1: An initial species containing several subarray position vectors d is randomly generated based on the subarray spacing requirements, where each position vector d is considered a chromosome in the GA algorithm or a particle in the PSO algorithm. Furthermore, each chromosome (particle) is given an initial velocity in the direction of the x and y axes.

Step2: The initial species are divided equally into M subspecies and optimized separately using the PSO algorithm.

Step3: Update the velocity and position vector of each particle using the following equation:

(41) $$\begin{array}{l} v_{k}(l+1)=av_{k}(l)+b_{1}r_{1}(d_{k-loc}-d_{k}(l))+b_{2}r_{2}(d_{glo}-d_{k}(l)),\\ d_{k}(l+1)=d_{k}(l)+v_{k}(l+1) \end{array}$$

where a is the inertia weight, $b_{1},b_{2}$ is the positive learning factor, $r_{1},r_{2}$ is a random number uniformly distributed between 0 and 1, $v_{k}(l)$ is the velocity vector of the particle after l iterations, $d_{glo}$ is the global optimal particle position vector, and $d_{k-loc}$ is the current optimal position vector of the k particle.

**Figure 5:**
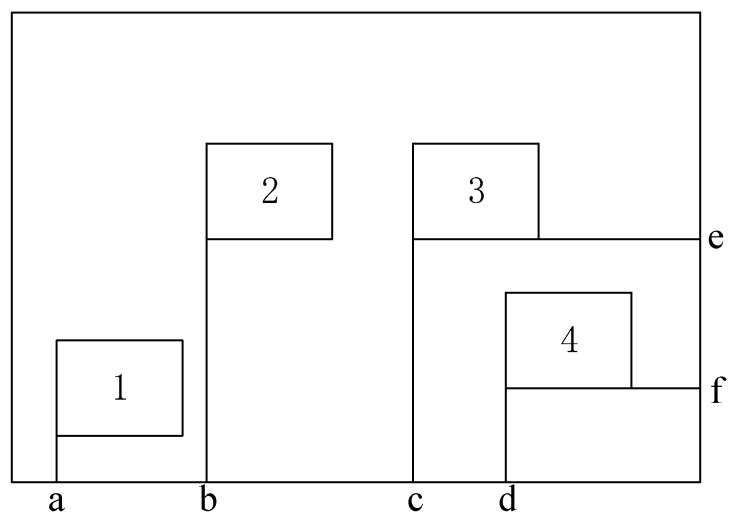
Schematic diagram of subarray overlap checking algorithm.

Step 4: Reasonability evaluation is the step which primarily determines whether the generated new subarray position vector d meets the constraints $0\leq x_{k}\leq L_{m},\;0\leq y_{k}\leq H_{m}$ and $\left|x_{k1}-x_{k2}\right|>\left(n_{1}-1\right)d\;or\;\left|y_{k1}-y_{k2}\right|>\left(n_{2}-1\right)d$, i.e., checking whether it is within the array range and whether the subarray elements overlap. First, the x coordinates of the subarrays are arrange from smallest to largest. Second, starting from the second subarray’s  x coordinates, the spacings with all previous subarrays x are calculated, and the subarray combination whose subarray spacing in the x-axis direction is smaller than $\left(n_{1}-1\right)d$ is recorded. Finally, it is determined whether the subarray combination $x_{n}$ in the y direction satisfies the constraint $\left|y_{k1}-y_{k2}\right|>\left(n_{2}-1\right)d$; if not, then the newly generated subarray positions do not meet the constraint. Subsequently, we need to regenerate coordinates for new subarray positions. As shown in Figure 5, suppose that there are four subarrays that need to be sparsely and optimally arranged, and only the spacing between subarray 3 and subarray 4 is in the  x axis direction $\left|cd\right|<\left(n_{1}-1\right)d$. Then, the spacing between subarray 3 and subarray 4 in the y axis direction needs to be compared with that in the *x* axis direction. Because $\left|ef\right|>\left(n_{2}-1\right)d$, this scheme meets the constraint that any two subarrays must not overlap.

Step 5: According to the equation of the fitness function (Equation (4)), the fitness value of each particle is calculated. Followed by storing them in the corresponding local optimal values fitness_local, updating the local position optimal vector $d_{k-loc}$, and identifying the global optimal particle $d_{glo}$ and its optimal fitness value fitness_local via comparison.

Step 6: If a new global optimal fitness value fitness_local is generated during the optimization process, then the counter for the number of consecutive non-updates is set to zero; otherwise, the counter is increased by one.

Step 7: If the number of consecutive non-updates exceeds the predefined threshold, then the PSO algorithm reaches a local optimal value. Then, the cycle is exited, and the optimal value is outputted. The criterion can be set to the extent that the local optimal value is not updated for $M_{1}$ consecutive times, and the local optimal condition is considered to be satisfied.

Step 8: When all subspecies are locally optimized by the PSO algorithm, all outputs can be synthesized into a new species.

Step 9: The new species is substituted into the GA and selection, crossover, and mutation operations are performed. The chromosome with the best fitness in each subspecies is directly selected, the remaining chromosomes are ranked from the smallest to the largest fitness values, and then the chromosome is selected for the next round using a roulette strategy. The crossover process is for exchanging a selected chromosome with another one in a randomly selected species for a randomly selected subarray position coordinates x or y. The mutation operation is a random change in the position coordinates  x or  y of a selected chromosome in a randomly selected subarray, resulting in a new chromosome.

Step 10: The reasonability evaluation in Step 4 is performed. The newly generated chromosome must meet the design requirements to proceed to the next step; otherwise, the previous step is repeated, and a new chromosome is regenerated.

Step 11: The fitness value of each chromosome within the species after crossover and mutation is calculated, and it is compared with the global optimal fitness values. If it is smaller than the current global optimal value, then the global optimal fitness value  fitness_local is updated and the counter is cleared; otherwise, the continuous non-update counter is increased by one.

Step 12: If the continuous non-update counter does not reach the maximum value $M_{2}$, then the iterative optimization using a GA is continued; otherwise, the optimal 20% chromosome is outputted to the selected species.

Step 13: The PSO algorithm is used again to perform local optimization on the result outputted by the GA algorithm, and the global optimal result is outputted if the optimal value is not updated for $M_{1}$ consecutive times. Note that the constraints must be checked for each operation (position update, crossover, and mutation) of the PSO algorithm and GA.

Step 14: The optimization process of the hybrid GA–PSO algorithm can be performed several times to output a set of several sparse antenna array schemes with similar fitness values, and comprehensive performance analysis and evaluation can be performed by combining the ambiguity functions of an FPMIMO radar based on a sparse antenna array of 2D subarrays provided in Equation (40). By identifying the advantages and disadvantages of the target distance and Doppler resolution according to the ambiguity function graph, we can determine the optimal array design.

Through analysis, the computational complexity of the proposed algorithm is mainly derived from two aspects: the cyclic iterations of an intelligent algorithm and the calculations of the radiation pattern during every iteration. Therefore, the computational complexity of the proposed algorithm is O((M + 1)$N_{pso}N_{ga}L_{1}L_{2}{Kn}_{1}n_{2}$), where $N_{pso}$ is the average iterations of PSO, $N_{ga}$ is the average iterations of the GA, and $L_{1}$ and $L_{2}$ are the number of sampling points for the azimuth and pitching, respectively. Compared with other algorithms, the computational complexity is relatively high, but it has no impact on the practical applications. Because the proposed algorithm is only used for designing the sparse antenna array for 2D subarrays, which can be completed with a supercomputer in an offline fashion, the FPMIMO radar only uses the optimization result of the proposed algorithm. Therefore, it is worthwhile to obtain a high-performance sparse array antenna by running this complex algorithm.

## 4. Simulation Results and Analysis

To verify the effectiveness of the proposed algorithm based on the GA–PSO algorithm and the ambiguity function for a sparse antenna array of 2D subarrays, in this section, we apply the algorithm to iteratively solve a sparse antenna array of subarrays. For the simulation experiment, the main simulation parameters are shown in Table 1, assuming that the radar signal frequency was $f_{c}=15\;\mathrm{GHz}$. Additionally, we needed to optimize an array of K=9 subarrays, each of which was a 2D uniform array of 9×9, with the internal elements being spaced at an interval of half the wavelength, and equipped with a sampling channel. The array was on a 2D plane which had a size of 0.45m×0.45m. In the optimization process, the uniform phase and magnitude distribution were used to obtain beamforming coefficients for each subarray. Moreover, the steering vector of each subarray was used as the weight vector.

During the simulation, simulation beam pointing was set to the $\left(0^{{}^{\circ}},\;0^{{}^{\circ}}\right)$ direction, and the subarray positioning accuracy was 0.1 mm. Each subarray was calibrated with the position of its reference array element, which is the element in the first row and first column of the subarray. With regard to the optimization objectives, the expected value of the PSLL was set to ${\mathrm{PSLL}}_{d}=-15\;\mathrm{dB}$; the upper limit of the error for the peak sidelobe was set to $P_{e}=5\;\mathrm{dB}$; the expected value of the main lobe width for the transmit radiation pattern was set to ${WB}_{d}=1.5^{{}^{\circ}}$; the upper limit of the error for the main lobe width was set to $W_{e}=0.5^{{}^{\circ}}$; the upper limit for the number of consecutive non-updates of the optimal fitness for the PSO algorithm and GA were set to $M_{1}=10$ and $M_{2}=30$, respectively; and the other parameters of the fitness function in the iterative operation were set to $\alpha_{1}=2$, $\alpha_{2}=1$, $\beta_{1}=1.5$, and $\beta_{2}=1$. The initial species size and number of loop iterations for the GA and PSO algorithm in the test were set to 100, and the species were divided equally into five subspecies. In the GA, the selection probability was set to 0.2; the crossover probability was set to 0.85; and the variance probability was set to 0.15. In the PSO algorithm, the inertia weight was set to a=0.8; the positive learning factors were $b_{1}=2$ and $b_{2}=2$; the angular resolution of the simulation experimental process was $0.2^{\circ }$; and the maximum motion distance of constrained particles in the x and y directions for each iteration process was 10*d*. In addition, the fitness functions of PSO and the GA were the same for the proposed approach in Equation (4), and the comparison result could prove the optimization performance of the proposed algorithm.

**Figure 6:**
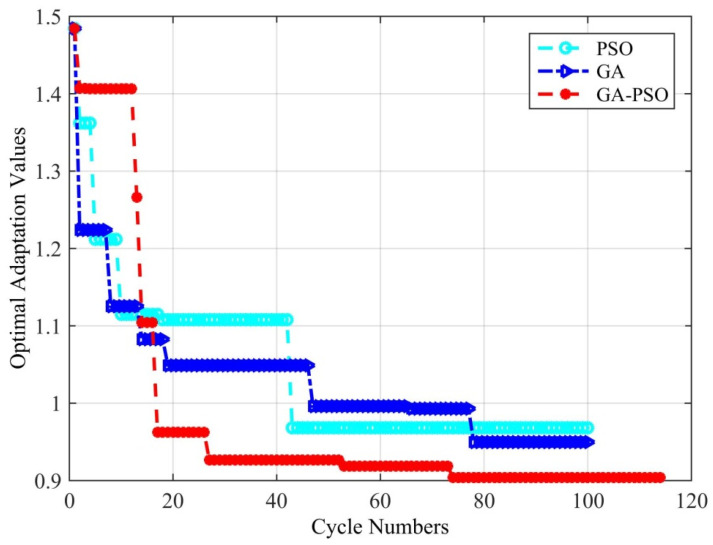
Simulation of the adaptation function of the optimized array algorithm.

**Figure 7:**
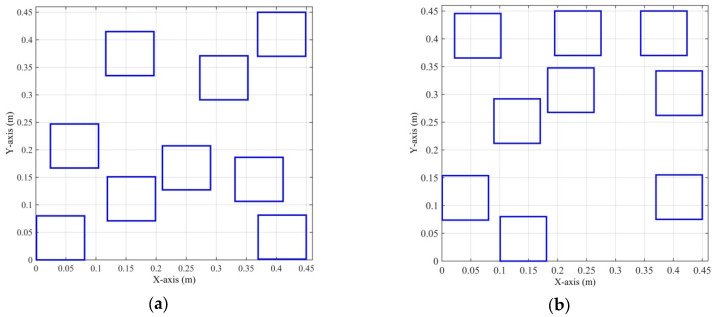
Experimental results of the sparse antenna arrays of subarrays. (**a**) Results of the first simulation experiment. (**b**) Results of the second simulation experiment.

Through the simulation experiments of two optimized arrays, the adaptation value change process for the first array optimization design cycle iteration process is shown in Figure 6. The proposed method achieved the best optimized array, with a PSLL of −15.14 dB, a main lobe width of $1.6^{{}^{\circ}}$, and an adaptation value of 1.0477. The optimized array is shown in Figure 7a, and the coordinates of its subarrays were

x = [0.2105, 0.0010, 0.0242, 0.1163, 0.1189, 0.2727, 0.3312, 0.3697, 0.3688],

and

y = [0.1273, 0.0000, 0.1670, 0.3349, 0.0710, 0.2909, 0.1064, 0.0014, 0.3700].

The PSLL for the second array optimization design was −15.36 dB, the main lobe width was $1.6^{{}^{\circ}}$, and the fitness value was 1.0182. The optimized array is shown in Figure 7b, and the coordinates of its subarrays were

x = [0.0220, 0.0006, 0.0900, 0.1008, 0.1829, 0.1950, 0.3436, 0.3700, 0.3700],

and

y = [0.3655, 0.0738, 0.2119, 0.0000, 0.2676, 0.3700, 0.3700, 0.0751, 0.2622].

**Figure 8:**
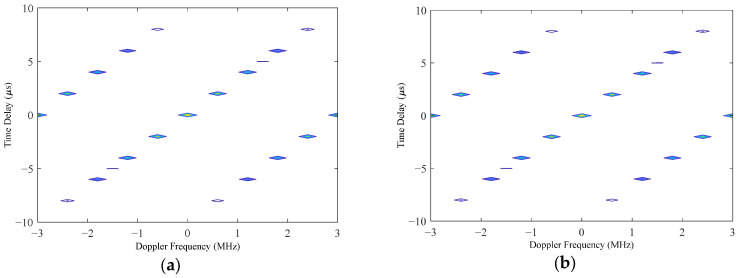
Contour plot of the 3D ambiguity graph, with the MF parameters being error-free. (**a**) Ambiguity function graph of the first optimized array. (**b**) Ambiguity function graph of the second optimized array.

**Figure 9:**
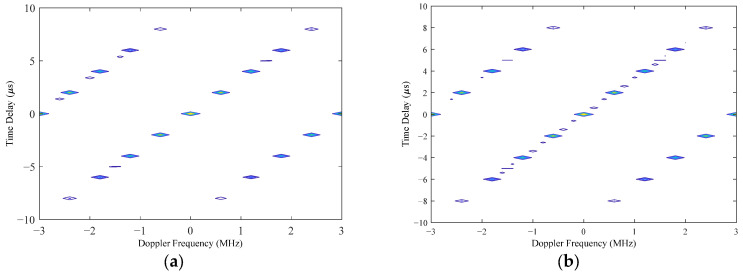
Contour plot of 3D ambiguity graph with a pointing error in the MF parameters. (**a**) Ambiguity function graph of the first optimized array. (**b**) Ambiguity function graph of the second optimized array.

**Figure 10:**
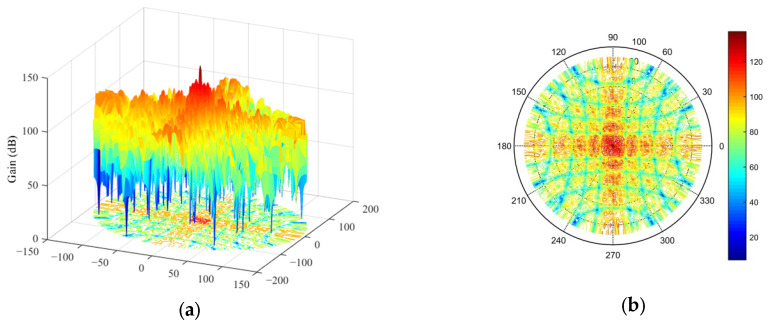
Transmit radiation pattern of the array optimized by the proposed algorithm. (**a**) Side view in polar coordinates. (**b**) Top view in polar coordinates.

According to the results of the two experiments, we can see that the main lobe width was $1.6^{{}^{\circ}}$ and the PSLL was below −15 dB; therefore, both optimization results met the predefined optimization objectives. To select an optimal array scheme from them, we could further analyze them by creating an ambiguity function graph. Figure 8a,b illustrates the contour plots of the ambiguity function for the first and second optimization results, respectively. According to these graphs, if there was no pointing error in the MF-processed transceiver beam, i.e., the transceiver beam and target location of each subarray were in the $\left(0^{{}^{\circ}},\;0^{{}^{\circ}}\right)$ direction, then the ambiguity function graphs of the two optimization schemes are almost the same. Meanwhile, when the MF-processed beam had a pointing error of $1^{{}^{\circ}}$ in the azimuth and elevation angles, i.e., the target space angle was $\left({-1}^{{}^{\circ}},\;{+1}^{{}^{\circ}}\right)$ while the MF-processed radar transceiver beam pointed in the $\left(0^{{}^{\circ}},\;0^{{}^{\circ}}\right)$ direction, then the contour plot of the ambiguity function is shown in Figure 9. Figure 9a shows that there was no significant change in the ambiguity function curve for the first optimized array, while the curve for the second optimized array shown in Figure 9b exhibits more spikes. This shows that the ambiguity function of the second optimized array was more affected by the pointing error of the MF-processed transceiver beam. Based on the results of the ambiguity function graph analysis, the first optimization result was selected as the final solution for the sparse antenna array, and the transmit beam pattern is shown in Figure 10. There was no grating lobe in the beam pattern, and the sidelobe gain was kept at a relatively low level, indicating that the optimization result achieved the predefined objectives.

**Figure 11:**
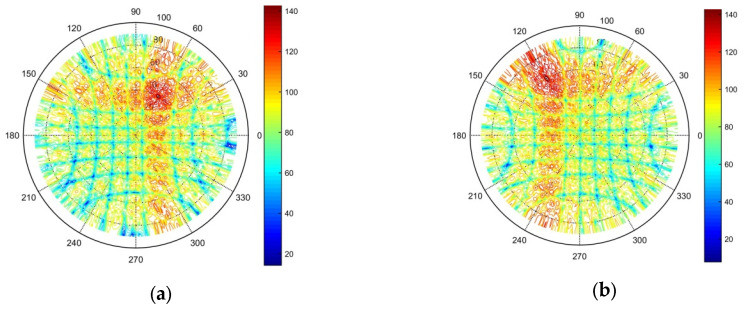

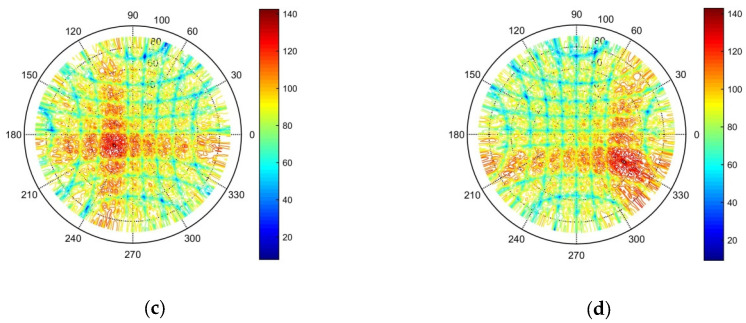
Simulation test of the transmit radiation pattern of the array optimized by the proposed algorithm. (**a**) Transmit beam at azimuth angle of $60^{{}^{\circ}}$ and elevation angle of $40^{{}^{\circ}}$. (**b**) Transmit beam at azimuth angle of $120^{{}^{\circ}}$ and elevation angle of $60^{{}^{\circ}}$. (**c**) Transmit beam at azimuth angle of $210^{{}^{\circ}}$ and elevation angle of $20^{{}^{\circ}}$. (**d**) Transmit beam at azimuth angle of $330^{{}^{\circ}}$ and elevation angle of $50^{{}^{\circ}}$.

To fully verify the effectiveness of the optimization results for the sparse antenna array of 2D subarrays, the validity of the array antenna’s transmit radiation pattern was tested, and the transmit beam gain was kept within the range of 0–150 dB to facilitate presentation of the results in polar coordinates. The simulation results are shown in Figure 11. Figure 11a shows the transmit radiation pattern when the transmit beam was pointing at an azimuth angle of $60^{{}^{\circ}}$ and elevation angle of $40^{{}^{\circ}}$; Figure 11b demonstrates the pattern when the transmit beam was pointing at an azimuth angle of $120^{{}^{\circ}}$ and elevation angle of $60^{{}^{\circ}}$; Figure 11c depicts the pattern when the transmitting beam was pointing at an azimuth angle of $210^{{}^{\circ}}$ and elevation angle of $20^{{}^{\circ}}$; and Figure 11d displays the pattern when the transmitting beam was pointing at an azimuth angle of $330^{{}^{\circ}}$ and elevation angle of $50^{{}^{\circ}}$. The simulation results show that by randomly pointing the transmit beam, the transmit radiation pattern only exhibited a peak in the beam pointing angle, and the grating lobe was not observed. Moreover, the side lobe gain was effectively suppressed, which fully proves the effectiveness of the optimized sparse antenna array of subarrays.

## 5. Conclusions

This study focused on the problem of determining an optimal design for a sparse antenna array of 2D subarrays. First, after determining the array aperture, number of subarrays, and subarray size, we defined the optimization objectives, i.e., eliminating the grating lobe and minimizing the PSLL and main lobe width. The spatial constraints in this problem are the following. The subarrays and their elements must be located within the spatial dimension of the array antenna, and the elements of any two subarrays must not overlap. The positions of the subarrays are optimized. By combining the GA and PSO algorithms and reducing the dependence of the optimization process on the initial species, an adaptation function with the objectives of minimizing the PSLL and main lobe width is established with the help of the relaxation function, and an optimized array of subarrays is proposed based on the GA–PSO hybrid optimization and ambiguity function. We realized global optimization by embedding the PSO algorithm into the GA and performing the selection, crossover, and variation operations of the GA. We reduced the time required for local optimization using the fast convergence of the PSO algorithm, which decreases the dependence of the GA algorithm on the initial species and aids in avoiding local optimal values. The proposed algorithm can adaptively adjust the number of optimization iterations according to the optimization results during optimization of the PSO algorithm and GA, which remarkably ensures the global optimization performance of the proposed algorithm. Because the array results of optimization calculation are often not unique, we propose combining the ambiguity function graphs of the array antenna to identify the final optimized sparse antenna array of 2D subarrays. Finally, the simulation test results prove the effectiveness of the algorithm proposed in this study.

## Figures and Tables

**Table 1:** Simulation main parameters.

Radar signal frequency: $f_{c}$	15 GHz
Number of subarray: K	9
Style of subarray	Two-dimensional uniform array
Antenna elements in each subarray	9×9
Prescribed area for placement	2D plane, size of 0.45 m × 0.45 m
Angel of beam pointing	$\left(0^{{}^{\circ}},0^{{}^{\circ}}\right)$
Expected value of the PSLL: PSLLd	−15 dB
Expected value of the main lobe width for the transmit radiation pattern: $WB_{d}$	1.5°

## Data Availability

The original contributions presented in this study are included in the article. Further inquiries can be directed to the corresponding author.
